# Pharmacologic interventions for postoperative nausea and vomiting after thyroidectomy: A protocol for systematic review and network meta-analysis

**DOI:** 10.1097/MD.0000000000014542

**Published:** 2019-02-15

**Authors:** Ye Jin Cho, Geun Joo Choi, Hyun Kang

**Affiliations:** Department of Anesthesiology and Pain Medicine, Chung-Ang University College of Medicine, Seoul, Republic of Korea.

**Keywords:** nausea, network meta-analysis, systematic review, thyroidectomy, vomiting

## Abstract

**Background:**

We aimed to perform a network meta-analysis (NMA) to quantify and rank the efficacy and safety of the pharmacologic interventions for prophylactic use for postoperative nausea and vomiting (PONV) in patients undergoing thyroidectomies.

**Methods:**

A systematic and comprehensive search will be performed using MEDLINE, EMBASE, the Cochrane Central Register of Controlled Trials (CENTRAL), and Google Scholar, beginning from their inceptions to February 2019. Only randomized clinical trials on the efficacy and safety of pharmacologic interventions for prophylactic use in patients undergoing thyroidectomies will be included.

The primary endpoints will be the incidences of postoperative nausea (PON), postoperative vomiting (POV), and PONV in the early, middle, late, and overall phases. The severity of PON, POV, and PONV; the use of rescue antiemetics; the incidence of complete response; and safety issues, such as headache, dizziness, drowsiness, and constipation, will be also assessed.

We will conduct both pairwise meta-analysis and NMA. We will use surface under the cumulative ranking curve (SUCRA) values and rankograms to present the hierarchy of pharmacologic interventions. A comparison-adjusted funnel plot will be used to assess the presence of small-study effects. The quality of the studies included will be assessed using the risk of bias tool 2.0. All statistical analyses will be performed using Stata SE version 15.0.

**Results:**

The results of this systematic review and NMA will be published in a peer-reviewed journal.

**Conclusion:**

This systematic review and NMA will provide a comprehensive and convincing evidence summary of prophylactic pharmacologic interventions for PONV after a thyroidectomy.

**Trial registration number:**

CRD42018100002.

## Abstract 2

Supplemental Digital Content is available in the text

## 1 Introduction

Postoperative nausea and vomiting (PONV) are the most common and unpleasant complications after anesthesia, among a list that includes aspiration pneumonia, fluid and electrolyte imbalances, and esophageal rupture.^[1–3]^ PONV even prolongs the average duration of a patient's visit in the post-anesthesia care unit and hospital, increases healthcare costs, and decreases patient satisfaction.^[4–6]^

In particular, vomiting after a thyroidectomy may increase the severity of postsurgical complications, such as surgical wound dehiscence, postoperative hemorrhage or neck hematoma, and, in the worst cases, airway obstruction due to hematoma.^[7,8]^

It has been reported that the overall incidence of PONV ranges from 22% to 52% after general anesthesia,^[9,10]^ whereas the incidence of PONV after thyroidectomy is between 60% and 84% when no prophylactic antiemetic is given.^[2,11,12]^ Numerous pharmacological interventions, including antihistamines, anticholinergics, dexamethasone, and multimodal approaches, have been studied for the prevention and treatment of PONV following thyroidectomy.^[8,13–17]^ Furthermore, a few systematic reviews have demonstrated the efficacy of dexamethasone for PONV after thyroidectomy.^[18–20]^ However, the relative efficacy and safety of pharmacological interventions still remain unknown.

Thus, we plan to conduct a systematic review and network meta-analysis (NMA) of published studies to comprehensively compare and rank the efficacy and safety of pharmacologic interventions for preventing PONV after thyroidectomy.

## 2 Methods

### 2.1 Protocol design and registration

Our protocol for the systematic review and NMA was developed by following the preferred reporting items for systematic review and meta-analysis protocols (PRISMA-P) statement.^[21]^ The protocol for this systematic review and NMA has been registered with the International Registration of Prospective Systematic reviews (PROSPERO network) and was assigned the registration number CRD42018100002, the record of which can be accessed on their website (https://www.crd.york.ac.uk/PROSPERO/display_record.php?RecordID=100002).

The present systematic review and meta-analysis will be conducted in accordance with the protocol recommended by the Cochrane Collaboration^[22]^ and will be presented according to the Preferred Reporting Items for Systematic Reviews and Meta-analyses guidelines for reporting NMA.^[23]^

### 2.2 Inclusion and exclusion criteria

#### 2.2.1 Types of studies

Peer-reviewed, randomized clinical studies will be eligible for inclusion. No language or date restrictions will be applied. Review articles, case reports, case series, letters to the editor, commentaries, proceedings, laboratory science studies, and any other non-relevant studies will be excluded from analysis.

#### 2.2.2 Population

Inclusion criteria for the study population will be as follows:

(1) patients receiving elective ambulatory thyroidectomy under general anesthesia, and
(2) patients who were given pharmacologic interventions that were then compared for PONV or pain control.

#### 2.2.3 Interventions and comparisons

Pharmacological interventions will include various kinds of antiemetics (ondansetron, ramosetron, palonosetron, granisetron, and dolasetron, among others); a steroid (dexamethasone); analgesics (NSAIDs, opioids, COX-2 inhibitors, acecaminophen, and lidocaine, among others); and hypnotics (midazolam, dexmedetomidine, and propofol, among others). Studies that compared non-pharmacological interventions, such as the administration of oxygen, the administration of fluids, acupuncture, or regional blocks, will be excluded.

#### 2.2.4 Outcomes

##### 2.2.4.1 Effectiveness

The primary endpoints will be the incidences of postoperative nausea (PON), postoperative vomiting (POV), and PONV in the early, middle, late, and overall phases. The severity of PON, POV, and PONV; the use of rescue antiemetics; and the incidence of complete response will be also assessed.

##### 2.2.4.2 Safety

Safety issues, including complications such as headache, dizziness, drowsiness, and constipation, will be assessed.

For the primary endpoints, the postoperative period will be divided into the early, middle, late, and overall phases. The early phase will be defined as 0 to 6 hours postoperatively, the middle phase as 6 to 24 hours postoperatively, and the late phase as after 24 hours postoperatively. If a study reported the data at multiple time points within the same phase, the data from the first time point will be selected as the outcome of interest (e.g., if the study reported data at 0 hour, 2 hours, 4 hours, and 6 hours postoperatively, we will only include the data at 0 hour as the early phase). If a study reported the data as falling within overlapping time points between phases, the data will be classified into the phase containing more of the overlapped range of time (e.g., if the study reported the data at 0 to 2 hours and 2 to 24 hours, we will define the data at 0 to 2 hours as the early phase and the data at 2 to 24 hours as the middle phase). To capture the maximum number of studies, any PON, POV, and PONV data from studies that do not mention a specific time point will be defined as data at the overall phase.

### 2.3 Information sources

#### 2.3.1 Electronic search

A search will be performed in MEDLINE, EMBASE, the Cochrane Central Register of Controlled Trials (CENTRAL), and Google scholar using search terms related to PONV. The search strategy, which includes a combination of free text, Medical Subject Headings (MeSH), and EMTREE terms, is outlined in the Appendix.

Additional relevant articles will be identified by scanning the reference lists of articles found during the original search and meta-analyses. Reference lists will be imported into Endnote software (Thompson Reuters, CA), and duplicate articles will be removed.

#### 2.3.2 Study selection

The titles and abstracts identified through the search strategy described above will be scanned independently by 2 of the authors of our study. To minimize data duplication as a result of multiple reporting, papers from the same author will be compared. For studies determined to be eligible based on the title or abstract, the full paper will be retrieved. All abstracts that cannot provide sufficient information regarding the eligibility criteria will be selected for full-text evaluation. Any potentially relevant studies chosen by at least 1 of the authors will be retrieved and evaluated in full-text versions. In the second phase, the same reviewers will independently evaluate the full-text articles and make their selection in accordance with the eligibility criteria.

Articles meeting the inclusion criteria will be assessed separately by 2 of the paper's authors, and any discrepancies will be resolved through discussion. In cases where an agreement cannot be reached, the dispute will be resolved with the help of a third investigator. A flow diagram for the search and selection process that follows the preferred reporting items for systematic reviews and meta-analysis (PRISMA) guidelines will be developed.

#### 2.3.3 Data extraction

All interrelated data from the included studies will be independently extracted, entered into a standardized form by 2 of the paper's authors (Cho YJ and Choi GJ), and then cross-checked. Any discrepancy will be resolved through discussion. If an agreement cannot be reached, the dispute will be resolved with the aid of a third investigator (Kang H).

The standardized extraction form includes the following items, and the following data will be extracted independently by 2 of the paper's authors:

(1) title;
(2) authors;
(3) name of journal;
(4) publication year;
(5) study design;
(6) registration of clinical trial;
(7) competing interests;
(8) country;
(9) risk of bias;
(10) number of patients in study;
(11) kinds and doses of drugs compared;
(12) sex of patients;
(13) age of patients;
(14) weight of patients;
(15) height of patients;
(16) duration of anesthesia;
(17) American Society of Anesthesiologists’ score of physical status;
(18) inclusion criteria;
(19) exclusion criteria;
(20) type of surgery;
(21) type of anesthesia;
(22) number of cases of PON, POV, and PONV overall and during the early, middle, and late postoperative phases;
(23) severity of PON, POV, and PONV;
(24) the need for rescue antiemetics;
(25) number of cases of complete response;
(26) the number of cases that reported headache, dizziness, drowsiness, or constipation.

If information is missing, an attempt will be made to contact the study authors to obtain the relevant information. If some data is presented as figures rather than numbers, the open source software Plot Digitizer (version 2.6.8; http://plotdigitizer.sourceforge.net) will be used to extract the numbers. For studies reporting the results from different doses in the same study, the groups will be combined in order to avoid a unit of analysis error.

The degree of agreement between the 2 independent data extractors (Kang H and Cho YJ) will be computed using kappa statistics to measure the difference between the observed and expected agreements between Kang H and Cho YJ; namely, whether they were at random or by chance only. Kappa values will be interpreted as follows:

(1) less than 0: less than chance agreement;
(2) 0.01 to 0.20: slight agreement;
(3) 0.21 to 0.40: fair agreement;
(4) 0.41 to 0.60: moderate agreement;
(5) 0.61 to 0.80: substantial agreement; and
(6) 0.8 to 0.99: almost perfect agreement.^[24]^

### 2.4 Study quality assessment

The quality of the studies will be independently assessed by 2 of the paper's authors (Cho YJ and Choi GJ), using the Revised Cochrane risk of bias tool for randomized trials (RoB 2.0).^[4]^ The risk of bias (ROB) will be evaluated by considering the following 5 potential sources of bias:

(1) bias arising from the randomization process;
(2) bias due to deviations from intended interventions;
(3) bias due to missing outcome data;
(4) bias in measurement of the outcome; and
(5) bias in selection of the reported result.

Then, we will evaluate an overall risk of bias judgment according to these domain-level judgments. The methodology for each domain will be graded as “Low risk of bias,” “Some concerns,” and “High risk of bias,” which reflects a low risk of bias, some concerns, and a high risk of bias, respectively.^[4]^

### 2.5 Statistical analysis

Ad-hoc tables will be designed to summarize the data from the included studies and show their key characteristics and any important questions related to the aim of this review. If a trial result is presented with 0 events in 1 group, then the event rate will be artificially inflated by adding 0.5. After the data have been extracted, reviewers will determine whether a meta-analysis is possible. For this, we will evaluate the heterogeneity and transitivity assumptions by examining the comparability of patient eligibility criteria, pertinent patient demographics, study design, and risk of bias (all degrees of bias versus removing “High risk of bias” arising from the randomization process and bias in measurement of the outcome) as potential treatment-effect modifiers across comparisons.^[25]^ We will note the methodological differences between studies that could influence outcome measurement as well as, any concerns related to the transitivity assumption or methodological heterogeneity.

Both a standard pairwise meta-analysis and an NMA will be conducted.

Initially, when at least 2 studies examine the same drugs, a pairwise meta-analysis will be conducted to generate summary estimates and to assess statistical heterogeneity across the included studies. Summary estimates will be reported as mean differences, standardized mean differences, or RRs, as appropriate, with corresponding 95% confidence intervals (CIs). Heterogeneity between studies will be assessed using the Cochran Q and the Higgins I^2^ statistics. A level of 10% significance (*P* <.10) in the Chi^2^ statistic or an I^2^ greater than 50% will be regarded as considerable heterogeneity, and the data will be analyzed using the Mantel–Haenszel random-effect model. Otherwise, we will apply the Mantel–Haenszel fixed-effect model.^[26]^

The publication bias will be assessed using Begg's funnel plot and the Egger test. If the funnel plot is asymmetrical *or* the *P* value is found to be <.1 by the Egger test, the presence of a publication bias will be considered, and trim and fill analyses will be performed.

When the treatment nodes form a connected network of evidence, we will perform an NMA. A multiple treatment comparison NMA is a generalization of meta-analysis methods that include both the direct randomized controlled trial (RCT) comparisons and also indirect comparisons of treatments. An NMA based on a frequentist framework will be performed with NMA graphical tools by Chaimani et al^[27]^ Given the clinical and methodological heterogeneity of the populations and methods among the included trials in NMAs, we will use the random-effects model in our primary analyses.

A network plot linking all the included analgesics will be formed to indicate the type of analgesics, the number of patients under different analgesics, and the amount of pair-wise comparisons. In the network plot, nodes will show the analgesic being compared, and edges will show the available direct comparisons between analgesics. Each drug, as well as each combination of drugs, will be treated as a node in this network. Nodes and edges will be weighted according to the number of patients and studies, respectively.

We will examine the consistency of the total network through both global and local tests of inconsistency. We will evaluate the global consistency assumption using the design-by-treatment interaction model.^[28]^ We will also evaluate each closed loop in the network in order to examine local inconsistency between the direct and indirect effect estimates for the same comparison. In each loop, we will estimate the inconsistency factor (IF) as the absolute difference (with 95% CI and a *z* test) between the direct and indirect estimates for each paired comparison in the loop. IF is the logarithm of the ratio of 2 odds ratios (RoR) from the direct and indirect evidence in the loop; RoR values close to 1 indicate that the 2 sources are in agreement.

We will also show the relative treatment effects between all active medications in ranked forest plots. Mean summary effects with CIs will be presented together with their predictive intervals (PrIs) to facilitate interpretation of the results in light of the magnitude of heterogeneity. PrIs provide an interval that is expected to encompass the estimate of a future study. We will not adjust for multiple comparisons in successive NMAs, as we are not interested in establishing the superiority or inferiority of particular comparisons.

A rankogram and cumulative ranking curve will be drawn for each analgesic. A rankogram plots the probabilities for treatments to assume any of the possible ranks. It is the probability that a given treatment ranks first, second, third, and so on, among all of the treatments evaluated in the NMA. We will use the surface under the cumulative ranking curve (SUCRA) values to present the hierarchy of interventions. SUCRA is a relative ranking measure that accounts for the uncertainty in treatment order, meaning it accounts for both the location and the variance of all relative treatment effects.^[29]^ A higher SUCRA value is regarded as a better result for an individual intervention. When ranking treatments, the closer the SUCRA value is to 100%, the higher the treatment ranking is relative to all of the other treatments.

We will test small study effects and publication bias using the comparison-adjusted funnel plot.^[30]^

A standard pairwise meta-analysis and NMA will first be performed based on data derived purely from studies for each drug, or for combinations of drugs, and re-analyzed according to the study design.

If clinical and methodological heterogeneity between study arms are found to be substantial, we will present the pairwise meta-analysis only. If the transitivity assumption cannot adequately be met, a descriptive summary of study findings will be presented. If inconsistency in the entire network or local inconsistency is suspected, we will conduct sensitivity analyses to evaluate the reason for the inconsistency as well as the influence of individual studies on the overall effect estimate by excluding 1 study at a time from the analysis. All statistical analyses will be performed using Stata SE version 15.0 (StataCorp, College Station, TX).

### 2.6 Evidence synthesis

Based on the results of the NMA for the RCTs, the overall quality of evidence for each outcome assessed will be rated using the guidelines developed by the Grading of Recommendations Assessment, Development, and Evaluation working group. These guidelines are designed to rate the quality of the effect estimates derived from an NMA and use sequential assessment of the evidence quality, followed by an assessment of the risk–benefit balance and a subsequent judgment on the strength of the recommendations.^[1]^ We will use a 4-step process:

(1) present direct and indirect treatment estimates (mean differences, standardized mean differences, or RRs with 95% CIs);
(2) rate the quality of direct and indirect treatment estimates;
(3) present the NMA estimates (pool of direct and indirect estimates, mean differences, standardized mean differences, or RRs with 95% CIs); and
(4) rate the quality of the NMA estimates.

### 2.7 Ethics and dissemination

#### 2.7.1 Ethical issues

This systematic review does not require an ethics approval or the need to obtain informed consent because there will be no direct contact with individual patients. Only previously published data will be included in the review.

#### 2.7.2 Publication plan

This systematic review will be published in a peer-reviewed journal and will be disseminated electronically and in print.

## 3 Discussion

PONV after thyroidectomy are the most common and distressing complications and increase hospital stays and health care costs.^[1,2]^

Although many strategies, including those with pharmacologic interventions, to prevent or reduce PONV have been extensively studied,^[8,31–34]^ the efficacy and safety of these interventions still remain unknown. Also, an NMA for pharmacologic interventions has not been studied until recently.

We designed this systematic review and NMA to compare and rank the efficacy and safety of pharmacologic interventions to reduce PONV in patients undergoing thyroidectomy. This study will search and merge all the current evidence and provide suggestions for clinical practice. To the best of our knowledge, this study will provide the first systematic review and NMA evaluating the efficacy and safety of pharmacologic interventions for prophylactic use for PONV in patients undergoing thyroidectomy. The result of this systematic review and meta-analysis will provide a comprehensive and objective assessment of pharmacologic interventions for preventing PONV, thus providing useful, convincing, and novel information and evidence for patients, anesthesiologists, surgeons, and policymakers.

## Author contributions

**Conceptualization:** Ye Jin Cho, Hyun Kang.

**Data curation:** Ye Jin Cho, Geun Joo Choi, Hyun Kang.

**Formal analysis:** Ye Jin Cho, Geun Joo Choi.

**Funding acquisition:** Hyun Kang.

**Investigation:** Ye Jin Cho, Choi GJ.

**Methodology:** Ye Jin Cho, Geun Joo Choi, Hyun Kang.

**Project administration:** Geun Joo Choi, Kang H.

**Resources:** Ye Jin Cho, Hyun Kang.

**Software:** Hyun Kang.

**Supervision:** Hyun Kang.

**Validation:** Geun Joo Choi.

**Writing – original draft:** Ye Jin Cho, Geun Joo Choi.

**Writing – review & editing:** Hyun Kang.

Hyun Kang orcid: 0000-0003-2844-5880.

## Supplementary Material

Supplemental Digital Content
